# Minimally invasive 1,444-nm Nd:YAG laser treatment for axillary bromhidrosis

**DOI:** 10.3389/fmed.2023.1034122

**Published:** 2023-02-06

**Authors:** Domenico Piccolo, Mohammed Hussein Mutlag, Laura Pieri, Irene Fusco, Claudio Conforti, Giuliana Crisman, Paolo Bonan

**Affiliations:** ^1^Skin Centers, Avezzano, Italy; ^2^Roma Clinic, Baghdad, Iraq; ^3^El.En. Group, Calenzano, Italy; ^4^Department of Dermatology and Venereology, Dermatology Clinic, Maggiore Hospital, University of Trieste, Trieste, Italy; ^5^Laser Cutaneous Cosmetic and Plastic Surgery Unit, Villa Donatello Clinic, Florence, Italy

**Keywords:** axillary bromhidrosis, axillary osmidrosis, axillary hyperhidrosis, Nd:YAG laser, subdermal coagulation, 1,444-nm laser

## Abstract

**Background:**

Axillary bromhidrosis is an apocrine glands hyperactivity disease.

**Methods:**

A total of 24 patients (15 men and 9 women) with axillary bromhidrosis underwent a laser procedure with a 1,444-nm Nd:YAG laser. Parameters evaluated in this study were as follows: the degree of malodor (T0, baseline; T30, after 1 month; and T180, after 6 months), postoperative pain, short-term decreased mobility (T1, after 1 day; T7, after 7 days; and T30, after 1 month), and overall satisfaction (T30, after 1 month and T180, after 6 months). A visual analog scale (VAS), from 0 to 10, was used to assess pain and decreased mobility, with lower values denoting less severity.

**Results:**

A total of 24 patients were followed up for 6 months after laser treatment. At baseline, all patients (100%) complained of a strong axillary malodor (mean degree of malodor at T0 = 2.0 ± 0.00). It decreased to 0.50 ± 0.64 at T30. At T180, the degree of malodor was 0.54 ± 0.57. Both T30 and T180 degrees of malodor significantly decreased from the baseline value (p < 0.01). The mean degree of patient satisfaction at T30 was 1.75 ± 0.52, and at T180, it was 1.67 ± 0.21. Among the 24 patients, eight complained of moderated pain 1 day after treatment. The pain subsided on day 7, except for two patients, with VAS = 1. Pain and mobility restrictions were in any case resolved within T30.

**Conclusion:**

Treatment with a 1,444-nm Nd:YAG laser for subdermal interstitial coagulation could be a less invasive and more effective option treatment for axillary bromhidrosis.

## 1. Introduction

Human sweat gland disorders are widespread and cause a considerable negative effect on social, psychological, and emotional wellbeing. It was found that the detrimental impacts of these ailments are equivalent to those of severe diseases such as rheumatoid arthritis, psoriasis, multiple sclerosis, and kidney failure (1). For this reason, it is essential to properly recognize and manage these disorders to guarantee the best patient care. While hyperhidrosis is caused by a sweat gland malfunctioning due to abnormally increased sweating (that greatly exceeds the human body’s thermoregulatory requirements), bromhidrosis (also known as osmidrosis, malodorous sweating, or body odor) is a chronic pathologic condition characterized by an excessive and extremely unpleasant body odor. Axillary bromhidrosis is a medical condition characterized by apocrine gland hyperactivity, and it is a common cause of consultation in dermatology. It is distinguished by a strong aggressive odor caused by the decomposition of bacteria in the apocrine secretions. Axillary odor control is a universal topic, and for this reason, the pathophysiology and etiology of axillary bromhidrosis have been extensively researched and described. The apocrine gland quantity, personal care, sex, age, emotions, hormonal level, ABCC11 genotype, concurrent hyperhidrosis, and disorders inducing bacterial overgrowth, such as intertrigo, diabetes, erythrasma, and obesity, all influence odor levels (2). Genetic histological examination of axillary tissue reveals that subjects with bromhidrosis have a greater number and dimensions of apocrine glands than control subjects (3). Skin surface bacteria decompose apocrine secretions into ammonia and short-chain fatty acids, which have distinct odors. Bacterial flora in the armpit area has been shown to convert non-odorous sweat precursors to more odorous volatile acids, resulting in a definite body odor (4). In most people with axillary bromhidrosis, familiarity was found due to the Mendelian traits of apocrine gland-related phenotypes (5). A variety of factors influence the level of intervention, including the severity of the symptoms, the patient’s personal choice, side effect tolerability, and consent to repeat the treatment several times (6). Improved hygiene, antiperspirant and antibacterial substances, botox, lasers, and, finally, surgery can be proposed, considering the patients’ expectations. Surgery has a great chance of success, but it also has an elevated complication rate, including skin necrosis, hematoma formation, and permanent scarring. Less invasive methods have frequently been linked to high recurrence rates. Recently, less invasive techniques improve the cure rate while avoiding the drawbacks of previous methods. In the present study, we illustrate our experience with the minimally invasive technique using a 1,444-nm Nd:YAG laser for subdermal coagulation treatment of axillary bromhidrosis.

## 2. Materials and methods

A total of 24 subjects (9 women and 15 men, 48 armpits) aged 19−57 (mean age, 35.4) years, with a BMI of 26.05 ± 2.05 kg m^–2^ and with axillary bromhidrosis, underwent a laser procedure with a 1,444-nm Nd:YAG laser (LipoAi; DEKA, Florence, Italy). Among them, 14 also had axillary hyperhidrosis. We excluded patients who were pregnant or lactating, as well as those who had a history of keloid formation. Based on the Helsinki Declaration, all subjects provided written informed consent to participate. The patients’ suitability to participate in the study was assessed. All subjects’ medical information was taken and recorded, a physical examination was carried out, and vital signs were controlled. Bromhidrosis was recognized when both the dermatologist and patient perceive the malodor. The preoperative degree of patient-assessed malodor was recorded for each subject. All treatments were done as outpatients with local anesthesia. To better define the hair-bearing area, which was delimited using a dermatological pen in the supine position with the arms abducted just before starting the procedure, all participants were instructed not to shave both axillae 1 week before the treatment. Following ordinary tumescent anesthesia, where an anesthetic consisting of a 0.4% lidocaine solution was injected into each side of the axillary fossa, two/four small holes (depending on the treatment area dimension) were made at the anterior and distal borders of each axilla with an 18−gauge needle. The handpiece is equipped with a very useful clip blockage passing through the laser handpiece and cannula. Moreover, the fiber insertion is protected by a fiber-protecting option, which is activated by changing the clamp position. Throughout the incision site, the cannula was introduced into the target layer of the dermal–subdermal junction. The transcutaneous guidance laser aiming beam was used to control where the cannula tip is during the whole procedure, monitoring both depth and position. A wider transcutaneous illumination area revealed the deeper fiber tip positioning. This allowed us to continuously check the correct position of the fiber during the procedure, which was fundamental to avoid any possible damage in deeper structures such as blood vessels or the brachial plexus. The laser was applied in a crisscross pattern with repeated cannulation. A foot switch was used to control the laser emission. Apocrine glands in the subcutaneous and dermal layers were destroyed by irradiation with a 600−μm fiber passing through the laser handpiece and cannula. The laser setting was as follows: pulse energy = 175 mJ and pulse rate = 40 Hz (power 7 W). The delivered energy varied from 1,800 to 2,300 J (mean delivered energy 2,092 J). We irradiated the laser once per 0.5−1.0 cm^2^ area within 1.5 s to avoid irreversible skin damage caused by excessive heat production after tissue-laser interaction. Cold packs were applied immediately after the laser treatment to the area to reduce the risk of skin heat injury. There was no need for sutures. The procedure took approximately 5 min on one side. After laser treatment, a compressive dressing was utilized for 24 h. We followed up with all patients on postoperative days 1, 7, 30, and 180 after the procedure.

Parameters evaluated during the study were as follows: the degree of malodor (T0, before treatment; T30, after 1 month; and T180, after 6 months), postoperative pain, short-term decreased mobility (T1, after 1 day; T7, after 7 days; and T30, after 1 month), and overall satisfaction (T30, after 1 month and T180 after 6 months). Malodor was graded as either absent (grade 0), fair (grade 1), or strong (grade 2). “Absent” means that nobody (neither the patient nor the physician, nor anyone nearby) was aware of the malodor. “Fair” indicates a not persistent malodor sometimes noticeable when the subject sweated. “Strong” implies that the patient and those around him or her were aware of the malodor. The satisfaction degree was classified into three scores: poor (grade 0), fair (grade 1), and good (grade 2) (7). The severity of malodor was the same on both sides of the axillary of the patient.

A visual analog scale (VAS) from 0 to 10 was used to assess the level of pain and decreased mobility, with lower scores indicating less severity. The occurrence of adverse reactions, such as burns, hematoma, infection, scarring, skin and fat tissue necrosis, sensory loss, seroma, allergic reaction, and anesthesia-related complications, was used to assess safety. In case symptoms of adverse reaction persisted, the patients were monitored until the healing was complete.

## 3. Results

**FIGURE 1:**
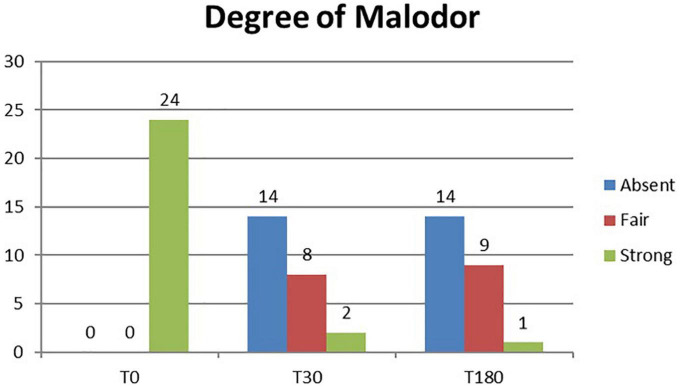
Histogram representation of the degree of malodor assessment results at baseline (T0) and study follow-up visits (T30 and T180).

**FIGURE 2:**
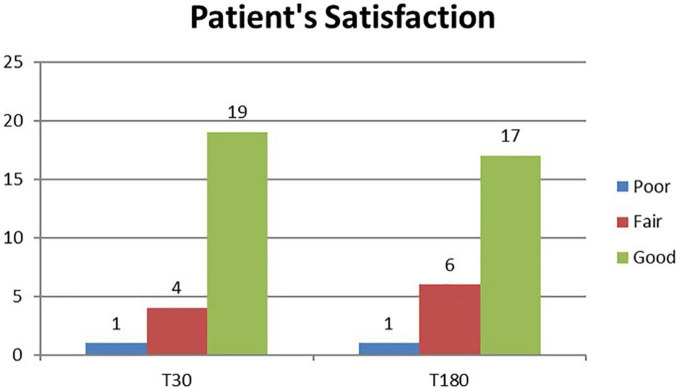
Histogram representation of patient satisfaction assessment results at study follow-up visits (T30 and T180).

A total of 24 patients were followed up for 6 months after laser treatment. At baseline, all patients (100%) complained of a strong axillary malodor. At T30 follow-up, malodor level was evaluated as absent in 14 patients (58%), fair in eight patients (33%), and strong in two patients (8%). At T180 follow-up, malodor level was evaluated as absent in 14 patients (58%), fair in nine patients (37%), and strong in one patient (9%) (Figure 1). The mean degree of malodor at baseline (T0) was 2.0 ± 0.00. It diminished to 0.50 ± 0.64 at 1 month (T30) after laser treatment. The level of malodor varied significantly at T0 and T30 (p < 0.001). Six months (T180) after the laser treatment, the malodor degree was marginally increased to 0.54 ± 0.57. However, it continued to remain significantly lower than the baseline value (p < 0.001), and the difference between the mean degree at T30 and T180 was not significant. Subject’s satisfaction was estimated (three-grade scale: 0 = poor, 1 = fair, and 2 = good). One month (T30) after the laser treatment, 19 patients (79%) reported good satisfaction, four patients (17%) reported fair satisfaction, and only one patient (4%) reported poor satisfaction. After 6 months (T180), 17 patients (71%) reported good satisfaction, six patients (25%) reported fair satisfaction, and one patient (4%) reported poor satisfaction (Figure 2). The mean degree of patient satisfaction 1 month after the laser surgery (T30) was 1.75 ± 0.52 and 6 months after laser surgery (T180) was 1.67 ± 0.21. The difference between the mean degree of satisfaction at T30 and T180 was not significant. A VAS scale (0−10) was used for documenting pain and mobility limitations after the procedure on day 1 (T1), day 7 (T7), and 1 month (T30) after surgery. On T1, 8 of the 24 patients complained of pain, and their mean VAS score was 1.63 ± 0.70. All of them no longer complained of any pain on day 7 except for two patients for whom the VAS score was, in any case, low (only 1). Pain and limitations in mobility were all resolved within 4 weeks of the treatment (T30). When compared to standard surgery, the side effects were mild. Twenty-eight of the 48 axillae treated (58%) had ecchymosis, which usually resolved within 1 or 2 weeks. Only one patient (4.2%) reported a shallow second-degree burn in one axilla only (2.1% of treated axillae). This burn was completely healed within 10 weeks using conservative methods with no scarring or contracture. The patient was followed up until the complete recovery. We noted no complications such as granuloma, seroma, dehiscence, or wound infection. During the clinical follow-up period, no skin necrosis or recurrences occurred. The procedure was successful, with only minor postoperative restrictions on patients’ social activities.

## 4. Discussion

**FIGURE 3:**
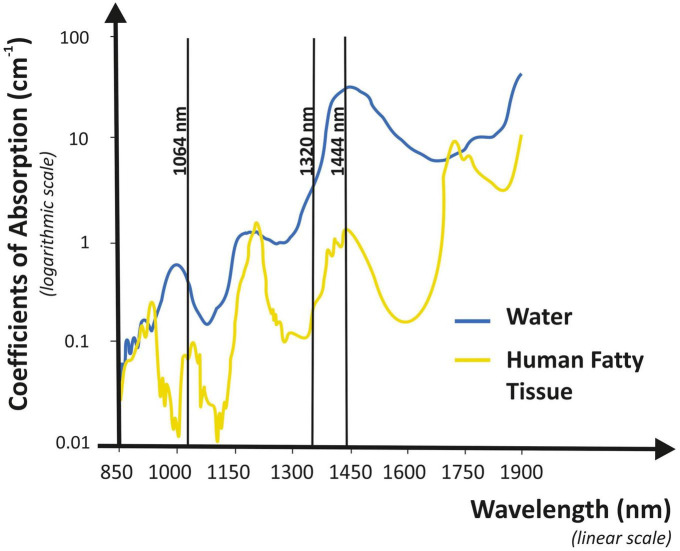
Graphical representation of absorption coefficient vs. wavelength for water and human fat. The 1,444-nm wavelength shows high selectivity characteristics and it is highly absorbed by human fat.

For axillary bromhidrosis, numerous therapeutic approaches have been developed, including both surgical and non-surgical options. Among surgical approaches, subdermal excision of the apocrine glands, suction-curettage technique, and subcutaneous scissor with micropore or endoscopic surgical treatment was generally performed. However, all these techniques require repeated treatments, induration, pain, and scarring in the armpits. Non-surgical methods are thought to have a higher frequency of recurrence than surgical procedures. Nevertheless, these techniques do not carry the risks of complications that can be associated with traditional surgery, such as axillary nerve plexus damage, strong postoperative discomfort, bleeding, and severely restricted mobility (8). For all of these factors, no consensus on the best surgical approach has been reached for treating axillary bromhidrosis and more and more patients continue to prefer minimally invasive procedures for treating it. This is the reason why new techniques have been developed. Patients who underwent liposuction for axillary bromhidrosis reported high satisfaction and quick recovery (9); Indeed, the overall complication rate was significantly lower than the percent complication rate seen with open surgical treatment. To improve the efficiency of liposuction for bromhidrosis, curettage can be combined with liposuction (10); when this combination was made (curettage and liposuction), almost all patients were satisfied with their outcomes. The use of both focused ultrasound and microwave technologies has been introduced. The first one acts on sweat glands that have a high water content, inducing cavitation which is followed by apocrine glands disruption (11, 12). Microwave therapy instead heats the layer between the skin and subcutaneous fat, resulting in sweat glands destruction (13, 14); the most common side effects were edema, redness, altered sensation in the skin of the treatment limb, and swelling outside the axilla. Lasers have also been adopted for axillary bromhidrosis treatment, dissolving fat and destroying apocrine glands. Following laser treatment, histopathological examination showed reduced density and considerable alterations in the apocrine glands (15). Axillary bromhidrosis has been treated with a variety of lasers. With all of them, the recovery time is short as well as the risk of adverse reactions is low, but the relapse frequency is higher than with traditional surgery. The CO_2_ laser was one of the first reported laser systems (16). Pulsed 1,064-nm Nd:YAG wavelength has achieved good results (17, 18). Unfortunately, its selective fat dissolution capability is quite low, so melting fat over a large area is difficult and time-consuming. Furthermore, researchers found that, among several wavelengths, 1,444 nm provides the highest degree of efficacy for fat tissue selective ablation and thermal confinement, permitting, in a short time, fat removal in different areas. When compared to other wavelengths, 1,444 nm is absorbed more strongly by the fat than the water. This wavelength has been shown to have a greater lipolytic effect in comparison with 1,064-nm (19) and 1,320-nm wavelengths, which mean a higher efficiency together with heat confinement with limited diffusion to surrounding tissue. In other words, 1,064 and 1,320 nm lasers need a greater amount of energy, three or two times, respectively, than that of the 1,444 nm laser to treat the same volume (20). The same study also observed that, because the fat is the main target, selective photothermolysis occurred at 1,444 nm because this wavelength matches up to a fat peak absorption, despite the fact that water absorption in the tissue is much higher (Figure 3). According to the scientific literature (8, 15, 21, 22), we believe that the 1,444-nm laser would specifically destroy apocrine glands in fatty tissues *via* a subdermal interstitial approach. After 6 months (T180), malodor was absent in 28 axillae (58%), fair in 18 axillae (33%), and poor in two axillae (4%). The majority of subjects treated (96%) were pleased with their outcomes for both efficacy and side effects. We also discovered that hyperhidrosis changes were related to bromhidrosis data. Although there are some differences between the present bromhidrosis study using a 1,444-nm laser and those mentioned previously, there was no significant difference in the clinical results among them. We tried to compare our findings to those of other treatment modalities such as ultrasound, microwave, and other Nd:YAG lasers (11–14, 17). The outcomes of most studies were evaluated in accordance with the patient satisfaction level, which ranged from 65 to 100%. It was challenging to compare these researches accurately because the majority of them employed various evaluation tools. To directly compare these modalities, more clinical research is required. We followed up on all patients on postoperative days 1, 7, 30, and 180, after the procedure and observed superficial second-degree burns on only one axilla (2.1%), which healed during conservative management. We found no complications, such as granuloma, seroma, wound infection, or dehiscence. During the clinical follow-up, no skin necrosis took place. The treatment using a 1,444-nm laser showed fewer significant adverse reactions in comparison with alternative approaches, such as liposuction or traditional surgery (9, 23). Main acute complications, such as postoperative pain and limited mobility, were less common in subjects undergoing laser therapy. According to our findings, except for mild ecchymosis and a skin crust induced by heat, laser treatment did not cause any long-term side effects. In any case, over-treatment and excessive heating can cause higher injury hazards or skin necrosis. Also, technical errors, such as skin injury produced by the fiber tip and incorrect target treatment, can cause necrosis of the epidermal layer. Therefore, laser operators should be properly trained to perform the procedure and must constantly monitor any changes in surface skin color. During the recovery process, the apocrine glands and fat tissue denatured by the laser were absorbed by surrounding tissues. As also assumed by Jeong et al. (8), the sweat glands that have not been completely denatured may re-engraft onto the tissue, potentially causing recurrence. In our experience, we did not record significant relapses during the study. This could be due to the limited follow-up of 6 months. Further investigations are necessary with a greater amount of patients and a longer follow-up to better clarify this aspect. Although not considered in our clinical study, liposuction and/or curettage can be combined with laser lipolysis (17), potentially resulting in greater effectiveness and better outcomes. Supplementary clinical trials are required to evaluate this possibility.

### 4.1. Study limitations

Further future studies will be required for long-term analyses with a larger patient sample size and the use of histological examination to better assess apocrine glands density reduction and skin necrosis development.

## 5. Conclusion

Treatment with a 1,444-nm Nd:YAG laser for subdermal interstitial coagulation could be a less invasive and more effective alternative option treatment for axillary bromhidrosis with benefits such as treatment speed, small wound size, quick recovery, unnoticeable scars, and return to normal regular lives. Additional studies with a greater amount of patients, a sham control group, and a longer follow-up are necessary to clarify some aspects left open by the present trial.

## Data availability statement

Data that support the study findings are available on request from the corresponding author (IF).

## Ethics statement

Ethical review and approval was not required for the study on human participants, in accordance with the local legislation and institutional requirements.

## Author contributions

DP, MM, LP, CC, GC, and PB: conceptualization, funding acquisition, methodology, and validation. LP: formal analysis. LP, MM, and IF: investigation. DP, MM, LP, CC, and GC: data curation. LP: writing—original draft preparation. LP and IF: writing—review and editing. DP, MM, LP, CC, GC, and PB: supervision. All authors read and agreed to the published version of the manuscript.
